# Prospective cohort study to evaluate the diagnostic values of biomarkers in blood, cerebrospinal fluid and magnetic resonance imaging for mild cognitive impairment, Alzheimer's disease and Parkinson's disease in Hispanics

**DOI:** 10.1002/alz.086130

**Published:** 2025-01-09

**Authors:** Josefina Fletcher, Eira Del Pilar Santamaría Cano, Anna Victoria Díaz Ramírez, Xenia E. Arosemena‐Aguero, Pahola Araujo, Ricardo Williams de Roux, Rainier Rodríguez Balaguer, Marilisa Díaz, Nelson Novarro‐Escudero, David Alberto Anguizola Tamayo, Lizeth Pinilla Aguilar, Julio Villarreal, Judy Mendoza, Verónica Billingslea, Jemila Juárez, Karen Courville, Sassil Zuridaday Pitti Degracia, Héctor Torres, Aron Benzadon, Bruno Hammerschlag, Ariel Saldaña, Lizzetha Yolanis Quinterio Girón, Dámaso Moisés Díaz Solís, Laura Mercedes Cerrud Castillo, David Dondis, Alexis Ariel Palacios Adames, Andrés Eduardo Bernales Crespo, Sofía Rodríguez Araña, Carolina Rodriguez, Natalia López, Diana C Oviedo, Maria B Carreira, Giselle Rangel, Alcibiades E Villarreal, Gabrielle B Britton

**Affiliations:** ^1^ Servicio de Neurología, CIDELAS‐CSS, Panamá, Panamá Panama; ^2^ Hospital Pacífica Salud, Panamá, Panamá Panama; ^3^ Hospital The Panama Clinic, Panamá, Panamá Panama; ^4^ Hospital Paitilla, Panamá, Panamá Panama; ^5^ Instituto de Investigaciones Científicas y Servicios de Alta Tecnología (INDICASAT AIP), Centro de Neurociencias, Panamá, Panamá Panama; ^6^ Universidad Santa María La Antigua, Panamá, Panamá Panama; ^7^ Sistema Nacional de Investigación (SNI), Panamá, Panamá Panama; ^8^ Hospital Dr. Gustavo Nelson Collado, Chitré, Herrera Panama; ^9^ Hospital Dr. Gustavo Nelson Collado, Panamá, Panamá Panama; ^10^ Servicio de Radiología, Hospital Paitilla, Panamá, Panamá Panama

## Abstract

**Background:**

By 2025, an estimated three‐quarters of the global population aged 60 and older will live in Latin American and Caribbean countries (LAC), leading to a rise in age‐related conditions. The main goal of the Panama Aging Research Initiative – Health Disparities (PARI‐HD) research program is to create a platform for multidisciplinary clinical studies that focus on mental health and its diseases such as age‐related cognitive decline. This platform would generate a large amount of clinical, neuropsychological, lifestyle, genetic and biomarker data following standardized research protocols. In this report we present the protocol of an ongoing clinical study within the PARI‐HD program. The main objective is to evaluate the diagnostic and prognostic value of biomarkers in blood, cerebrospinal fluid and magnetic resonance imaging for mild cognitive impairment (MCI), Alzheimer's disease (AD) and Parkinson's disease (PD) in Panamanian older adults (≥ 65 years of age). We report a description of the overall design of the research study and the demographic characteristics of the research cohort enrolled to date.

**Method:**

The study is descriptive and prospective, and for each participant we obtain informed consent, demographic data on medical history and functional status and neuropsychological evaluations. CSF, MRI and fasting blood samples are obtained. Participants are divided into the following diagnostic groups: MCI, AD, PD, and without cognitive impairment.

**Result:**

To date, 72 persons have completed the baseline clinical evaluation: MCI (n=28), AD (n=4), Parkinson's disease (n=9), and without cognitive impairment (n=31). The participants have also completed the following study activities: MRI scans (n=38), lumbar punctures (n=23), blood samples (n=66), and cognitive tests (n=70).

**Conclusion:**

The aim of this protocol is to enroll 120 participants across the four diagnostic groups, to highlight the critical role of precise brain imaging and CSF biomarkers in disease detection. Our study is the first of its kind in Panama and one of few in the LAC region, setting a milestone in exploring CSF protein analysis and MRI techniques associated with MCI, PD and AD in Hispanic/Latino individuals aged 65 and older.

